# Report on Dipthentic Croup

**Published:** 1853-09

**Authors:** Morgan Snyder


### ECLECTIC DEPARTMENT.

### *Report on Diptheritic Croup.*

BY MORGAN SNYDER, M. D.

In accordance with a resolution adopted by this society at its annual meeting, held in February, 1851, I submit the following report:

Among the many forms of disease which medical men are called upon to attend, few, if any are calculated to excite a deeper interest or a stronger feeling of sympathy, in behalf of those afflicted, than diptheritic croup. Generally sudden in its attack, terrible in its nature, and producing great fatality, it becomes invested with peculir interest to all to cultivate and improve our knowledge of such a disease. To mitigate its terrors and lessen its fatality, is an object worthy of the attention of every physician.

To deny that the disease and a rational treatment of it is much better understood now than it was half a century ago, or at the time of Bretonneau, would cast an unworthy and unjust reflection upon the many able writers, whose researches and essays, since that time, have contributed largely to point out its true nature, and a more satisfactory management. It must be confessed, however, from the records of cases, and our own experience, and observation, that the methods of treatment among physicians, at the preseat time, vary greatly, are unsatisfactory to themselves, and that nearly all the severe cases prove fatal.

It is not designed by this paper to enter into a detail or discussion of laryngeal or tracheal forms of croupal disease, its sole object is to give a brief account of that form of the disease attacking the tonsil, glands or fauces, and extending into the larynx; that form of the disease called by Bretonneau, diptherite, and which, as seeming to me more expressive of its whole character, I have termed diptheritic croup.

The cases upon which the few observations about to be offered are founded, are about thirty in number, having occurred within the last five years, and embrace, with very few exceptions, all the cases of croupal disease noticed within that period. It is worthy of remark, too, that these cases have not appeared, at any time, in the form of an epidemic. They have been as occasional as other ordinary diseases, and have not afforded any evidence of being infectious. The majority have occurred in the spring, summer, and autumn months; all have occurred in children between the ages of one and twelve years.

Diptherite is an inflammation of the throat and fauces, extending into the larynx, producing an exudation of lymph or mucous or albuminous matter. The parts are painful, red, swollen, and sometimes ulcerated: patches of the exudated matter gather upon the surface and clog up the laryngeal passage, or a false membrane is formed, producing all the symptoms and sufferings of simple croup; and it generally terminates in death in a few hours or days, if not overcome by treatment. It usually occurs suddenly in children or adults, where previous constitutional derangement has existed, and where the patient is laboring under some form of inflammation of the mouth, tonsil glands, or fauces, during sudden changes of temperature, or in damp and cold weather, by the sudden influence of cold upon the system. This inflammation extends to the air passages; is attended with the characteristic exudation, and constitutes the most formidable and fatal disease to which childhood is subject.

Although this form of disease does not always follow, yet it doubtless is to be apprehended in all cases of inflammation of the mucous membrane of the mouth, whether in infants at the breast laboring under aphthous disease, in cases of muguet, in those having enlargement of the tonsil glands, or inflammation of those parts in adults. It also assumes the same form and course when those parts are attacked as a sequence of diseases of the skin, as in scarlet fever, measles and small pox; or it may attack suddenly the throat and fauces, and extend rapidly into the larynx.

In endeavoring to obtain from books a knowledge of this disease, as the cases referred to have come under my notice, it has seemed to me that a considerable confusion as to its true knowledge exists, at least a discrepancy in its description is very evident, whatever may be the cause of it.

In their work on diseases of children, Evanson and Maunsell say "another form of exudation differing in its character and seat, is to be observed in children affected with inflammation of the pharynx, tonsils or soft palate. Children are subject to common inflammation of these parts, like adults, not differing in nature or mode of treatment, except as far as danger arises from the greater narrowness of the passage and proximity of the rima glottidis; and so the greater risk of suffocation, hence, however, much promptness in the application of antiphlogistic means is necessary. But in children, in particular, we are liable to find inflammation of these parts throwing out a false membrane, and this, whether the inflammation show a tendency to sloughing or not; the presence of this membrane, which appears to be an exudation of the true lymph, is always a source of danger from its tendency to spread in the larynx, and constitutes the disease known as diptherite, as described by Bretonneau, and spoken of by him as croup."

Dr. Green says, "there exists a form of exudative inflammation, which Mr. Bretonneau calls diptherite, or the croup of adults, but which is not exactly identical with the croupal inflammation of children, the same parts however, are affected in both diseases, and they both end in the effusion of plastic lymph; but true croup ordinarily commences with catarrhal symptoms, is more sthenic in its nature, and is confined in its attacks to children, and persons before the age of puberty, whilst the above form of disease commences with pain, redness, and swelling of the tonsils and back of the throat generally, and attacks, moreover, individuals of all ages, but those especially who have become debilitated by other diseases."

Dr. Copland regards this form of disease as a complication of croup, but has given a very accurate description of its symptoms, progress and termination.

This form of disease frequently appears also as an epidemic. Several writers have distinctly described it aside from Bretonneau; among those of remote periods, are Hamilton, Deslandes, Bourgeios, Ferriar and Hosack, besides numerous others of more recent date. Dr. Brown, of Haverford, West England, met with an epidemic in 1851, of two hundred cases.

In regard to the pathological nature of this affection, authors are not agreed, and I will not enter into a recital of the various theories offered. Fever, laborious breathing, croupal cough, swelling, tenderness and redness of the parts, with occasional ulceration, and the *exudation,* are its characteristics; and indicate an inflammation of the follicular glands, the mucous surface and areolar tissue beneath.—Delicate and complicated as these parts are, and being copiously supplied with blood vessels, it seems to me that the ordinary laws of inflammation readily account for the changes produced; as in other surfaces, the inflammation unchecked spreads continuously and according to its severity and the different textures involved, depending somewhat upon constitutional vigor, or susceptibility of disease, will we see manifested the various grades of action and results, which are observed in these parts, such as simple inflammation, exudation into the areolar tissue, or upon the surface of the membrane, or both combined; or abrasions of the mucous membrane, or ulcerations extending into the areolar tissue.

The cases which have fallen under my notice have indicated the different manifestations of diseased action, and these have afforded a very good index of their severity and danger. Some have been connected with, or seemed to occur in consequence of previous inflammation of the mouth, and have been attended with ulceration of the mouth and tonsil glands; others had disease of the tonsil glands only; whilst in several the attack seemed sudden and general upon the tonsils and fauces. Those attended with ulceration have always appeared to me milder and less rapid in their progress; and when the exudation was found not to extend beyond the tonsil glands, or slightly upon the fauces, have yielded to treatment. Those, on the other hand where the attacks have been more severe, when the inflammation extended rapidly, or when the patient was not seen in the first onset of the disease, and when it had extended to the epiglottis and larynx, or as far as the parts were visible, have all proved fatal but one.

A detail of the appearance, progress, treatment and termination of the last four cases which have been under my care, will serve to give the general character of them of all.

On the 24th of August, 1852, I was called to visit the son of Mr.

R., aged 6 years. He had suffered with enlargement of the tonsil glands, during the previous three years, and was much worse at times on slight exposure or sudden changes of weather. At this visit I found he had considerable fever, difficulty of breathing and croupal cough, which had been worse than in former attacks during the two previous days. Upon examination of the throat, I found the glands large, red, and an exudation upon their surface forming gray colored patches, and the same state of the fauces and parts as far as they could be seen; the breathing was very much obstructed, and the collection of mucus large; the tongue was white, and considerable febrile excitement existed. I prescribed an emetic of ipecac, which relieved him considerably, put him on powders of ipecac and calomel every three hours; ordered warm fomentations to the neck, and sponged the throat with a solution of nit. silv., 20 grs. to the oz., which produced relief in breathing after each application a short time; the calomel was continued, the strength of the nit. silv. increased to 60 grs. to the oz., and applied three times daily, rest was procured by pulv. Dover or elixir paregoric, and nutritious food administered. He gradually grew worse and expired on the 5th day after he came under treatment.

On the 26th September, 1852, I saw a child of Mr. B., aged 2 years and 6 months, I was informed that it had been subject to occasional attacks of croupal symptoms, that they had usually subsided by giving an emetic and other simple remedies; I found the pulse quickened, skin hot and dry, tongue white, obstructed breathing, and other symptoms characteristic of croupal disease. The tonsils were slightly enlarged, of a scarlet red color, covered with patches of gray exudation, and the same appearance of the fauces and parts as far as visible, with slightly offensive breath. I prescribed an emetic of ipecac, and ordered warm fomentations to the neck, after which gave powders of calomel and ipecac every two hours, applied to the parts a solation of nit. silv. 40 grs. to the oz., and had this application made three times in the twenty-four hours. Sleep was produced with elixir paregoric; this treatment was continued about 48 hours, when from an abatement of the symptoms, I entertained strong hopes of recovery. At this stage the mother conceived the idea that a less energetic or milder treatment would answer, and contrary to my urgent advice, confided its care to an aged lady as nurse. In about twelve hours the symptoms returned with increased severity, and the child expired on the 29th, three days after my first visit.

On the 29th October, 1852, I visited the child of Mr. M., aged 2 years, and found the parents greatly alarmed by the fear of croup.—The little patient had suffered the few previous days with looseness of the bowels; the skin was hot and dry, pulse full and hard; there was difficulty of breathing, restlessness, and a dry, shrill, croupal cough; the tongue was coated, and several ulcerated spots existed in the mouth; the tonsil glands were enlarged, of a deep red color, ulcerated, and a considerable mucous exudation upon their surface. I prescribed for this child an emetic of ipecac, and had hot fomentations applied to the neck; gave calomel 2 grs. rhubarb 4 grs., to be admintered twice in twenty hours, for the purpose of acting as a laxative on the bowels, and a decoction of rhubarb and soda, to be given occasionally during the day, and ordered the mouth and throat frequently sponged with a solution of bi-borate of soda, and once daily with a solution of nit. silv., 60 grs. to the oz.; after the bowels were sufficiently acted upon, gave small doses cal. and pulv. Dov. every 3 hours.—This treatment was persevered in, and the child recovered in about six days.

On the 25th November, 1852, a child of Mr. W., aged two years and two months, which had been afflicted with enlarged tonsil glands, was taken ill suddenly, with croupal cough, laborious breathing, fever, &c.; the mouth had a few ulcerated spots, the tonsil glands were large, red, and ulcerated. Mild antiphlogistic remedies, with cal. and ipecac, were prescribed, fomentations ordered to the neck, and a solution of bi-borate of soda ordered to sponge the mouth and throat every two hours. On the 26th, breathing and cough rather worse, and a considerable exudation on the tonsils and fauces; same remedies continued, and the throat sponged with a solution of nit. silv., 60 grs. to the oz., night and morning and intermediately every two hours with the solution of borax. Under this treatment the symptoms subsided on the third day and the patient was quite well at the expiration of ten days.

The fatal cases were mostly of the character of the first two mentioned when the attacks were severe, and the force of the disease exerted suddenly upon the tonsils and fauces, when some hours had elapsed between the attack and the time of my visit, where they were attended by a copious exudation and a tumefaction of the parts as far as visible, and evidently extended into the larynx. On the other hand those which have recovered were milder in their character, and the exudation slight or confined to a small surface. Sixteen of the cases of this character have recovered, fourteen have proved fatal.

One of the severest cases before referred to occurred in February, 1850. The son of J. W., aged 6 years, and it is worthy of notice on account of the result, the mouth and tonsil glands were ulcerated, the exudation extending over all the parts of the fauces visible, and the greatest possible diffiulty in breathing existed for some three days.—This child resisted effectually every attempt to apply the sponge to the fauces. The treatment consisted in occasional emetics of ipecac and powders of calomel, ipecac and camphor, every three hours, anodynes, hot fomentations to the neck, and blistering on the nape; this child recovered, and by the use of hot fomentations, and the calomel powders only, has been relieved of two attacks since that time.

*Treatment.*—The treatment and management of this disease, demands prompt and decisive action on the part of the physician, as well as judgment in the use of remedies. I can only suggest such a course, as has appeared to me from my knowledge and observations in the cases treated, the most efficacious.

I regard antimonials, drastic cathartics, and all depleting and depressing remedies, as not only injurious, but as having a tendency to produce a fatal result.

In some of the cases which first came under my care, I tried emetics of sulphate of copper, have used calomel and alum in combination, and resorted to some depletion, have blistered the throat, over the upper part of the sternum and on the nape of the neck, but without satisfactory results.

Emetics of ipecac, given early after the attack, have answered extremely well, as preparatory to other means, and also during its continuance, to expel the collection of mucus gathering on the fauces and in the larynx. They not only give great temporary relief, but serve in many instances to protract life, and thereby assist in giving time for the influence of other more potent remedies. Calomel administered early, so as to evacuate the bowels, and afterward in smaller quantities, for the purpose of procuring its constitutional influence; elix. paragoric, or pulv. doveri, to allay spasmodic action of the muscles of the larynx, or to procure rest; hot fomentations to the throat, warm pediluvia, and sponging the fauces and larynx with solutions of bi-borate of soda, or nit. silv., or nit. acid, constitute the main remedies to be relied upon in the treatment of this disease, according to our present knowledge.

When the pulse becomes frequent, sweating takes place, and other indications of prostration and sinking of the vital powers exist, brandy, wine, carb. ammonia, quinine, and other stimulants, and tonics are useful, but they will not arrest the fatal termination, unless the local difficulty is first overcome.

In relation to the use of nitrate of silver, I hope a few remarks will not be inappropriate. Several physicians have said to me they have tried this application, but the results did not meet their expectations, and they had no confidence in it. Upon inquiry, I have ascertained that it is used in strength varying from 10 grains to the ounce of water up to 60 or 80 grains, and that some use it in the solid form. It has seemed to me that the unsatisfactory results pertaining to the use of this article are to be accounted for from the mode of using it, and a want of proper attention to the effects produced by it upon the parts upon which it is applied.

In its application, two distinct objects should be held in view; the one to obtain a perceptible effect upon the mucous membrane, and yet not *destroy its surface*; the other to reach as far as possible the whole of the diseased surface; the sponge should cause an altered appearance of the surface of the parts, or according to Prof. Bennett, "produce a white precipitate, in the form of a molecular membrane, which defends for a time the tender mucous surface or irritable ulcer, and leaves the passage free for the act of respiration." From 40 to 80 grains to the ounce will usually answer this intention. Weaker solutions will not always produce this effect; and although I have used them, have thought the application useless, if not irritating; large collections of mucus have seemed to me also to prevent a favorable action, and I have been led to adopt the plan, to cleanse out the parts first with cold water.

In the milder cases, I have used the nit. silv. daily, and ordered frequent sponging with a solution of bi-borate of soda. In the severer forms two and three applications have been made in the twenty-four hours; if the disease does not yield it might be used more frequently, or the acids resorted to.

Dr. Rockwell, of the city of New York, has informed me that he had succeeded in cases of this disease by producing speedy mercurialization by means of a warm vapor bath. His plan is, for a child from two to four years old, to take about one scruple of cinnabar, place it upon a red hot brick, and cover this and the patient for a short time with a sheet or large bag; the mercurial vapor in this way is breathed and absorbed, and a few applications, in from two to twenty-four hours, produce the constitutional effect, and with it a relief from the croupal difficulty. From my experience of the good effects of mercury in this disease, I can not doubt the propriety of such a plan of treatment.

I have found great difficulty in some cases in applying the sponge satisfactorily to the fauces of children, and believe that on account of their resistance, and the smallness of the passage, it is almost impossible to reach beyond the epiglottis; on this account, the cases, in which the disease has extended considerably, especially into the larynx, so commonly prove fatal. In such cases I believe the principal hope of recovery rests upon the rapid induction of mercurial action in the system.

I have not resorted to tracheotomy in any case, and have no experience of its utility or success.

I cannot omit to urge upon physicians the great propriety of examining carefully, the state of the tonsil glands and fauces, in all cases where croupal symptoms exist. This is not only important in view of obtaining a correct knowledge of the disease, and the aid of local measures in its commencement, when it may not have extended beyond the tonsils or fauces, but also of avoiding the fatal error of regarding it as the laryngeal croup of authors, and pursuing accordingly an active depletory treatment.

Notwithstanding the most energetic and skillful management of this disease, and the very best selection of remedies, and care in their application, most of the severe cases will prove fatal, yet if by calling the attention of the profession to a form of disease, which in our latitude is becoming common and prevalent, I could excite an interest which would result in greater care in its management, more study and investigation of its character, or a better or more successful form of treatment, so that one more interesting child or youth could be saved from its deadly grasp, or one more mother spared the pangs of witnessing the death of her offspring so horrible and revolting, I would feel amply gratified for the labor attending the performance of this duty.—*From Trans. Med. Soc., of State of New York.*

